# Prevalence estimates of human immunodeficiency virus (HIV) infection among visceral leishmaniasis infected people in Northwest Ethiopia: a systematic review and meta-analysis

**DOI:** 10.1186/s12879-020-4935-x

**Published:** 2020-03-12

**Authors:** Mehdi Mohebali, Yonas Yimam

**Affiliations:** Department of Medical Parasitology and Mycology, School of Public Health, Tehran University of Medical Sciences, Tehran, Iran

**Keywords:** Visceral leishmaniasis, HIV, Ethiopia, Systematic review, Meta-analysis

## Abstract

**Background:**

In Ethiopia, by the end of 2018, an estimated 690,000 people are infected with HIV and the annual cases of Visceral Leishmaniasis (VL) is estimated to be between 4000 and 5000 with over 3.2 million people are at risk. Northwest Ethiopia accounts for over 60% cases of VL in the country. Prevalence of HIV infection among VL infected people in Ethiopia has not yet been synthesized. Therefore, we aimed to estimate the pooled prevalence of HIV infection among VL infected people in Northwest Ethiopia with the hope that it would guide the development of a more robust and cost-effective intervention strategies.

**Methods:**

In this systematic review and meta-analysis, we searched six international databases: PubMed, Ovid MEDLINE®, Embase, Scopus, Google Scholar, and ProQuest Dissertations & Theses. We also searched reference lists of included studies and Ethiopian universities electronic thesis and dissertation repositories. The search was performed until June 30,2019. Funnel plot symmetry visualization confirmed by Egger’s regression asymmetry test and Begg rank correlation methods was used to assess publication bias. Pooled prevalence estimate was calculated using Der Simonian and Laird’s random Effects model. We went further to perform univariate meta-regression and subgroup analysis to identify a possible sources of heterogeneity among the studies. STATA software (version 14, Texas, USA) was used for analysis.

**Results:**

From 1286 citations identified by our search, 19 relevant studies with 5355 VL infected individuals were included in this meta-analysis. The pooled prevalence of HIV infection among VL infected individuals in Northwest Ethiopia was 24% (95%CI: 17–30%). The result of sensitivity analysis demonstrated that the pooled prevalence estimate was robust and not one-study dependent. The pooled prevalence estimate of HIV infection among VL infected people in Northwest Ethiopia ranged from 20.88% (95%CI: 15.91–25.86) to 24.86% (95%CI: 18.57–31.14) after a single study was deleted.

**Conclusions:**

The burden of HIV infection in people infected with VL in Northwest Ethiopia is considerably high. Integrating HIV/AIDS surveillance among VL infected people would improve case detection as well as prevention and control of disease spread.

## Background

Leishmaniasis is a plethora of clinical syndromes caused by *Leishmania* parasite, spread by the bites of infected female sandflies [1–3]. Leishmaniasis is widespread in tropical and subtropical areas, reported in more than 89 countries including Africa, United States of America (USA), the Mediterranean region, Europe and South-East Asia [4, 5]. Leishmaniasis is principally a zoonotic disease, except for two species; *Leishmania donovani* and *Leishmania tropica*. There are over 53 *Leishmania* species, 90 sandflies and 70 different animal hosts that are involved in the zoonotic transmission cycles [6, 7]. Annually, 0.7 to 1 million new cases occur, of which 26,500 to 65,000 succumb to the disease globally [7]. Leishmaniasis can present in various clinical forms, but the three most common presentations are visceral, cutaneous, and mucocutaneous forms [2, 7].

Visceral leishmaniasis (VL), commonly known as kala-azar disease, affects the reticuloendothelial system, including the spleen, liver and bone marrow, which is the most severe and lethal form, especially if not detected and treated early [8, 9]. The causative agents of VL vary in different geographical regions; *Leishmania donovani* in Africa and India, *Leishmania infantum* in the Middle East and Mediterranean regions, and *Leishmania chagasi* in Southern Europe [10]. In VL infected people, the clinical signs and symptoms are irregular bouts of fever, remarkable weight loss, splenomegaly, hepatomegaly, and pancytopenia [11]. There has been a substantial decline in the global incidence of VL, from 200,000–400,000 in 2012 to 50,000–90,000 in 2017, and 94% of the new cases (20,792 out of 22,145) are restricted to 7 countries such as Brazil, Ethiopia, India, Kenya, Somalia, South Sudan and Sudan [5, 7]. The first case of VL in Ethiopia was reported in 1942 in the lower Omo plains of the Southwestern region [12]. Now, it has spread to the Northwest, Northeast, and Ethio-Dijibuti border [5, 13]. The transmission of VL in Ethiopia is mainly anthroponotic due to *Leishmania donovani* with an annual rate of 5000 new cases [13, 14].

HIV and VL concomitant infection (HIV-VL) has been recognized as an emerging challenge in global public health [15]. In 1985, the first case of HIV and VL concurrent infection was recorded [16]. In 1997, the number of patients concurrently infected with VL and HIV reached a peak; however, in between 1998 and 2001, HIV-VL co-infection was observed to be stable. After 2001, simultaneous infection of HIV and VL has shown declining trends in many parts of the world [17]. However, HIV coinfection in people infected with VL remains high in East Africa, and this region bears the highest loads of HIV infection in VL infected people in the world [18]. To date, HIV and VL concomitant infection is reported from 35 countries [19, 20]. In VL endemic areas, where many cases of VL are asymptomatic, HIV infection exacerbates the progression of asymptomatic VL towards VL disease and atypical sites. The existence of HIV-VL coinfection accelerates the risk of progression of active VL by 100–2320 times and further complicates diagnosis, response to treatment, and transmission [21].

Ethiopia, located in East Africa, bears the second biggest VL burden in Sub-Saharan Africa’s next to Sudan and shares Sudanese border [14]. HIV prevalence in Ethiopia has reduced from 1.5% in 2011 to 1.1% in 2015 [22]. Though the burden of HIV infection is diminishing in the general Ethiopian population, the prevalence of HIV infection in VL patient is considerably high [23, 24]. According to various study reports on co-occurrence of HIV and VL in Ethiopia, the prevalence is considerably inconsistent and heterogeneous, ranging from 0.82% [25] to 67.5% [26]. Besides, to the best of our knowledge, there was no previous study that estimates the pooled prevalence of HIV coinfection in people infected with VL in Ethiopia; hence, this systematic review and meta-analysis was carried out. We hoped that our findings would guide the development of a sound, economically feasible, reliable, and sustainable intervention program to curtail the burden of the infections.

## Methods

This systematic review and meta-analysis was carried out in accordance with PRISMA (Preferred Reporting Items for Systematic Reviews and Meta-Analysis) guidelines [27]. Studies that reported HIV infection prevalence in VL infected people and conducted in Northwest Ethiopia were considered for this systematic review and Meta-analysis.

### Search strategy

For this systematic review and meta-analysis, we did a comprehensive search of PubMed, Ovid MEDLINE®, Embase, Scopus, Google Scholar, and ProQuest Dissertations & Theses, supplemented by search of reference lists of included studies and Ethiopian universities electronic thesis and dissertation repositories until June 30, 2019, when the last search was conducted. All searches were performed using the Medical Subject Heading (MeSH) term and keywords. We used the following search terms: “visceral leishmaniasis,” “Leishmaniasis, Visceral,” “visceral leishmaniasis hiv” and “Ethiopia.” The MeSH terms and keywords were used individually or in conjunction using Boolean logic operators such as “AND” or “OR”. For instance, the following search strategy was used in all fields of PubMed: (((visceral leishmaniasis OR Leishmaniasis, Visceral OR visceral leishmaniasis hiv)) AND Ethiopia). All the searched literature was imported to EndNote X7 software (Thompson Reuter, CA, USA) for management of retrieved studies. Eligible studies were selected for final analysis in two phases. In the first phase, study reports were scrutinized by titles and abstract to include the studies in the full-text assessment. In the second phase, full-text articles were evaluated as per the inclusion/exclusion criteria. Any inconsistency in the selection process of the studies by the two authors was settled by agreement and consensus.

### Inclusion and exclusion criteria

For this systematic review and meta-analysis, we included cross-sectional, retrospective time series, case-control and randomized control trial studies that reported the prevalence of HIV coinfection among people infected with VL in Northwest Ethiopia regardless of the study period, and publication status. Studies that reported sample size and the prevalence of HIV in VL patients were included in order to estimate the pooled prevalence of HIV infection in VL infected people in Northwest Ethiopia. We omitted studies that reported only VL prevalence, studies conducted outside of Northwest regions of Ethiopia, case reports of HIV and VL coinfection, reviews and studies that did not report sample size. For simplicity and clarity, only studies published in the English language were included.

### Data extraction and quality assessment

For data retrieval, we developed an extraction format and the following information extracted: name of author/s, year of publication, study period, study area, study design, sample size, the prevalence of HIV infection in VL infected people and types of diagnostic methods used for VL. In this review, the primary outcome was prevalence of HIV infection among VL infected people. HIV coinfection in people infected with VL was calculated by dividing number of individuals with HIV coinfection by the number of VL infected individuals. Quality assessment of the included studies was done strictly following Hoy 2012 risk of bias assessment tool [28]. The tool consisted of 10 items, and then the overall risk of bias assessment was rated based on the number of the high risk of bias per study: low (≤2), moderate (3–4), and high (≥5).

### Data analysis

For data analysis, we used STATA statistical software (version 14, Texas, USA). The inverse variance weight in the meta-analysis of fixed-effects is sub-optimal when operating on binary data with low prevalence. Thus, we transformed point prevalence estimate of studies by variance stabilizing double arcsine transformation by the following formula: t = arcsin (sqrt (r/(n + 1))) + arcsin(sqrt ((r + 1)/(n + 1))), where t = transformed prevalence, r = positive numbers, and *n* = sample size; se(t) = sqrt(1/(n + 0.5)), where se = standard error and the back transformation to a proportion is done using: *p* = (sin(t/2))^2^. The measure of variability among studies (heterogeneity) was evaluated using Cochran’s Q-test (X^2^), and *p*-value lower than 0.05 indicates the presence of heterogeneity. Moran’s I^2^ (inconsistency) was used to evaluate the percentage of variation in the prevalence estimate due to heterogeneity. Inconsistency can be interpreted as low, medium, or high when I^2^ values are 25, 50%, or 75%, respectively. Tau-square (Tau2) statistic was also applied to measure variations between studies. Funnel plot symmetry visualization followed by Egger’s regression asymmetry test and Begg rank correlation methods were used to detect the presence of publication bias. The point prevalence estimate of each study with a 95% confidence interval was used to estimate pooled prevalence using the Der Simonian and Laird’s random effects model. Furthermore, to assess the possible sources of variation, univariate meta-regression was conducted based on sample size and year of publications. Sensitivity analysis was performed by step-by-step omitting of a single study to evaluate the robustness of pooled prevalence estimate.

## Results

**Fig. 1:**
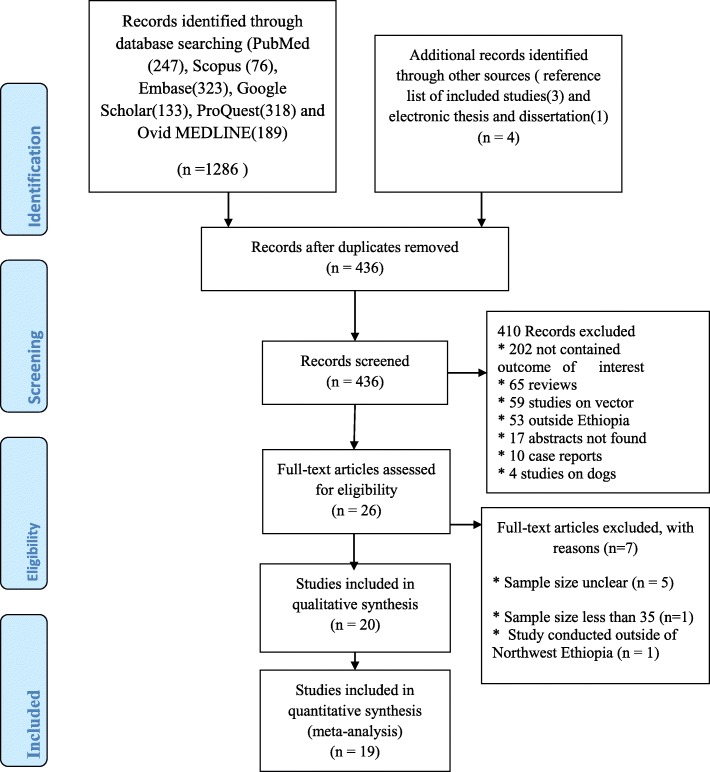
PRISMA flowchart showing the process of study selection for pooled prevalence estimate of HIV infection among VL infected people in Northwest Ethiopia, 2001 to 2019

The process of article selection is as described in the figure (Fig. 1). In the initial step of our search, a total of 1290 articles were recovered from six databases: PubMed, Scopus, Embase, Google Scholar, ProQuest, and Ovid MEDLINE® and manual searching of literature. Of these, 854 were excluded due to duplication. The remaining 436 articles were screened for titles and abstracts, and 410 studies excluded further. Among 26 studies included for full-text assessment, seven studies were excluded with reasons. Finally, 19 studies were included for meta-analysis.

### Characteristics of the included studies in meta-analysis

Characteristics of all the 19 studies included in this systematic review and meta-analysis are presented in Table 1. Regarding geographical region of the studies, 100% (19/19) studies were conducted in Northwest Ethiopia. A total of 5355 VL infected study participants were included in this systematic review and meta-analysis. The included studies were carried out from 2001 to 2019. Concerning study designs, 10 studies were facility-based retrospective, four studies were facility-based cross-sectional, three studies were randomized control trials, 1 study was prospective, and 1 study was facility-based case-control study. The smallest and highest sample sizes of the included studies were 52 and 595, respectively. With regards to the risk of bias assessment of included studies, 16 studies had low risk, and 3 had a medium risk.

**Table 1:** General characteristics of studies included in this meta-analysis

S.N.	Author(s) and year of publication	Study Area	Study period	No. of VL infected people	No. of individuals with HIV coinfection	Prevalence of HIV coinfection (%)	Study de sign	Risk of bias assessment
1	Alemayehu et al., 2017 [29]	Northwest	2016	462	82	17.75	Facility-based cross-sectional	Low risk
2	Bantie et al., 2014 [30]	Northwest	2013	109	33	30.3	Facility-based case-control	Low risk
3	Beshah, 2011 [31]	Northwest	2005–2009	111	41	37	Facility-based retrospective	Medium risk
4	Endris et al., 2014 [32]	Northwest	2012	83	13	15.7	Facility-based Cross-sectional	Low risk
5	Diro et al., 2019 [33]	Northwest	2014–2015	536	81	15.11	Randomized control trial	Low risk
6	Diro et al., 2015 [34]	Northwest	2012–2013	570	36	6.3	Facility-based retrospective	Low risk
7	Diro et al., 2015 [25]	Northwest	2011–2012	122	1	0.82	Facility-based prospective study	Medium risk
8	Hailu et al., 2010 [35]	Northwest	2008–2009	52	13	25	Facility-based retrospective	Medium risk
9	Herrero et al., 2009 [36]	Northwest	2005–2007	298	50	16.77	Facility-based retrospective	Low risk
10	Hurissa et al., 2010 [24]	Northwest	2006–2008	241	92	38.17	Facility-based retrospective	Low risk
11	Lyons et al., 2010 [37]	Northwest	1998–2000	213	49	23	Facility-based retrospective	Low risk
12	Mengesha et al., 2007 [38]	Northwest	2012	403	42	10.4	Facility-based cross-sectional	Low risk
13	Mengistu and Ayele, 2007 [23]	Northwest	1999–2004	212	87	41	Facility-based retrospective	Low risk
14	Ritmeijer et al., 2011 [39]	Northwest	2004	375	107	28.53	Randomized control trial	Low risk
15	Ritmeijer et al., 2006 [26]	Northwest	2007–2009	289	195	67.5	Facility-based retrospective	Low risk
16	Ritmeijer et al., 2001 [40]	Northwest	1998–1999	147	27	18.6	Randomized control trial	Low risk
17	Ter Hors et al., 2009 [41]	Northwest	2006–2007	128	44	34.4	Facility-based cross-sectional	Low risk
18	Welay et al., 2007 [42]	Northwest	210–2013	595	49	8.2	Facility-based retrospective	Low risk
19	Yimer et al., 2014 [43]	North, Amhara	2013	409	74	18.1	Facility-based cross-sectional	Low risk

### Heterogeneity and publication bias

**Fig. 2:**
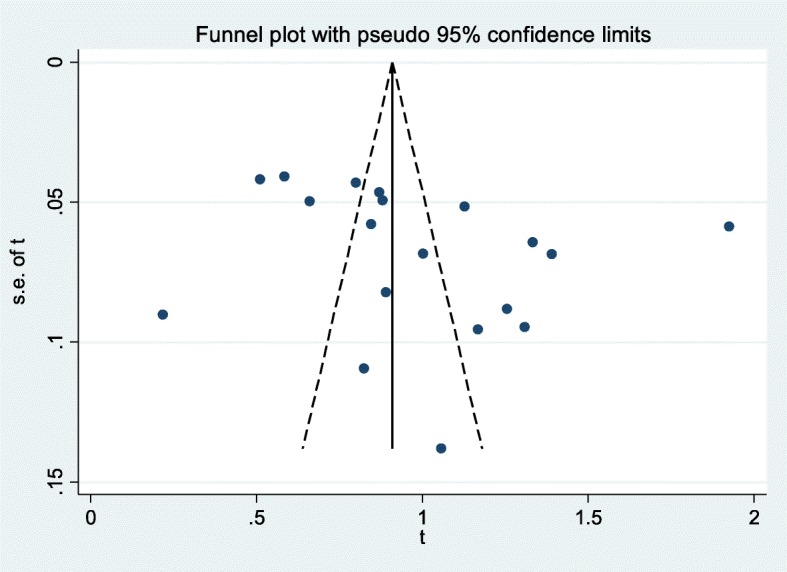
Meta funnel plot presentation of prevalence estimate of HIV coinfection among VL infected people in Northwest Ethiopia, 2001 to 2019. Abbreviation: t: arcsine transformed prevalence estimate of HIV coinfection among VL infected individuals and se of t, standard error of t

The funnel plot symmetry visual inspection was used to assess the presence of publication bias qualitatively, and shows the absence of publication bias (Fig. 2). Besides, the absence of publication bias was statistically confirmed by Egger’s weighted regression test (bias coefficient (B) = 6.76, (95%CI = − 2.78–16.13%; *p* = 0.15). Therefore, we did not perform Trim and Fill analysis to adjust the final pooled prevalence estimate. Heterogeneity analysis indicated the occurrence of high variations among studies (I^2^ = 98.1%, *p* < 0.001). Consequently, Der Simonian and Laird’s random effects model was used to estimate pooled prevalence.

### Pooled prevalence estimate

**Fig. 3:**
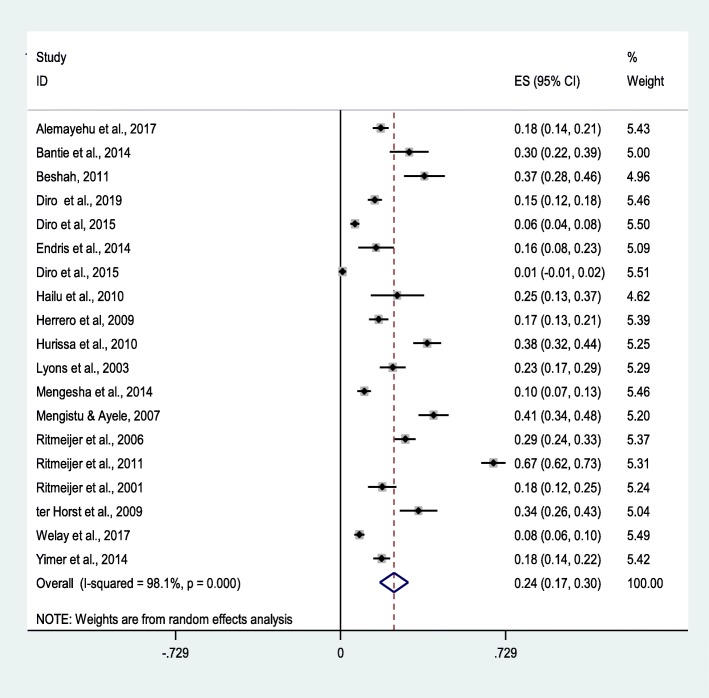
Forest plot showing the pooled prevalence of HIV infection among VL infected people in Northwest Ethiopia, 2001 to 2019

A total of 5355 VL infected study participants were included in this meta-analysis, 1116 were coinfected with HIV. The prevalence of each of the studies included in this systematic review ranges from 1% (95%CI: − 1-2%) to 67.5%(95% CI: 62–73%) with pooled prevalence estimate of 24% (95% CI: 17–30%); I^2^ = 98.1%, *p* < 0.001) (Fig. 3). Due to high heterogeneity among studies, univariate meta-regression analysis was employed to evaluate the sources of variations based on sample size and year of publications. According to the univariate meta-regression analysis, the year of publication showed a statistically insignificant relationship with the prevalence of HIV coinfection in VL infected people (*p* = 0.094). Also, the prevalence of HIV infection in VL infected people was not shown statistically significant association with sample size (*p* = 0.17).

### Sensitivity analysis

**Fig. 4:**
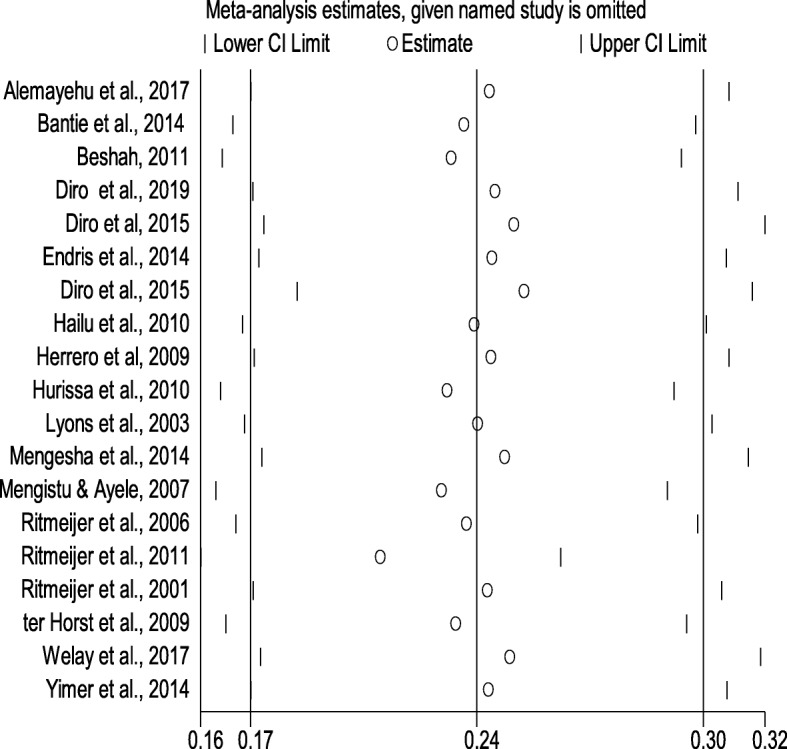
Sensitivity analysis of included studies to determine the pooled prevalence estimate of HIV infection among VL infected people in Northwest Ethiopia, 2001 to 2019

Sensitivity analysis of HIV coinfection in people infected with VL in Northwest Ethiopia was performed using a random-effect model. Sensitivity analysis was carried out by excluding each study step-by-step from the meta-analysis and comparing point prevalence estimate before and after removing a single study. Accordingly, removing a single study did not alter the pooled prevalence estimate considerably, with sensitivity analysis ranging from 20.88% (when [26] was removed) and 24.86% (when [25] was removed) (Fig. 4).

### Meta-regression

**Fig. 5:**
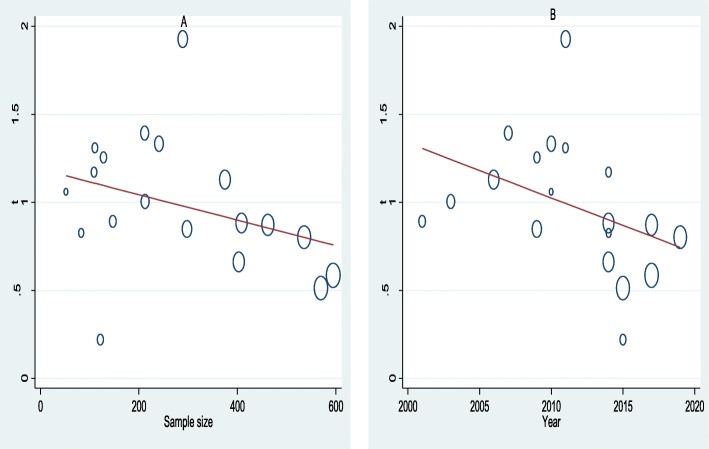
Meta-regression plot of prevalence of HIV coinfection in VL infected people in Northwest Ethiopia based on sample size (A) and year of publication (B), 2001 to 2019

Univariate meta-regression analysis was performed to assess the trends of HIV coinfection in people infected with VL based on sample size and year of publications. Meta-regression between the prevalence of HIV infection in VL infected people and year of publication showed a statistically insignificant declining trends of HIV coinfection in people infected with VL from 2001 to 2019 (B = −.045, *p* = 0.094). Similarly, there was no statistically significant correlation between the prevalence of HIV infection in VL infected people and sample size, although there was a small reduction in the prevalence with rising sample size (B = −.00072, *p* = 0.17) (Fig. 5).

## Discussion

This meta-analysis was the first of its kind in Ethiopia. To determine pooled prevalence estimates of HIV infection among VL infected people, we used 19 studies that conducted from 2001 to 2019. Concomitant infection of HIV and VL has a paramount implication in clinical manifestation, therapeutic response, diagnosis, and epidemiology of VL [44]. Thus, this review was undertaken to estimate pooled prevalence of HIV infection in VL infected people in Northwest Ethiopia that would provide significant information in the control of HIV and VL coinfection threats.

In this systematic review and meta-analysis, the pooled prevalence estimates of HIV infection in VL infected people in Northwest Ethiopia was 24% (95% CI: 17–30%). The finding of the present study is higher than the prevalence rate of HIV and VL concomitant infection in East African countries like South Sudan (2.5%) [18] and Sudan (3.6–9.4%) [18] and higher than what was reported in West African country like Burkina Faso (14.3%) [45]. Furthermore, the result of this study was considerably higher than studies undertaken in South-East Asian and Latin American countries, like India (2.18%), Nepal (5.7%) and Brazil (2%) [45]. Such discrepancies in the magnitude of HIV and VL coinfection in different parts of the world may be attributed to intricate differences in host factors, socio-economic factors, parasite factors, and vector factors.

In this study, heterogeneity among studies was very high, with the lowest and highest prevalence of HIV coinfection in VL infected people was 1%(95% CI: − 1-2%) and 67%(95% CI: 62–73%), respectively. The lowest prevalence of HIV coinfection was observed in a study exclusively included pediatric visceral leishmaniasis population, whereas the highest prevalence was observed in adult visceral leismaniasis population. This disparity could be explained by the substantially high number of people living with HIV at and above the age of 16 (650,000) compared to the number of people living with HIV between the ages of 0 and 14 (36,000) [46].

The pooled prevalence of HIV coinfection in VL infected people in Northwest Ethiopia is substantially high. Northwest Ethiopia, bordering Sudan, is an important endemic area of visceral leishmaniasis that accounts for over 60% cases of VL in the country [47, 48]. The region is renowned by high agricultural activity, where sesame, cotton, and sorghum are mainly commercially cultivated. This farming activity draws a large annual influx of about 200,000 seasonal male migrant workers mostly from neighboring Amhara and Tigray region, where VL is not endemic. These male migrant workers may stay months to years making them susceptible to acquiring VL [49–51]. This, in turn, increases the arrival of female commercial sex workers in the region, which may add to the high prevalence of HIV infection in VL infected people in the area [21].

The prevalence of HIV infection in VL infected people didn’t show a declining trend based on the year of publication of the studies, from 2001 to 2019 (B = −.0312, *p* = 0.094). This could be explained by the fact that still consistently high numbers of high HIV cases are reported in neighboring areas of the Tigray and Amhara regional states where the present study was conducted, and inconsistence implementation of prevention and control measures [52]. Nevertheless, the prevalence of HIV/AIDS in VL patients (18%) is still substantially higher than the general population, as reported in recent studies [29], pinpointing the need for coordinated and integrated control approach, especially in the endemic areas.

Some of these study weaknesses are: First, significant heterogeneity has been noted among studies. Thus, the pooled prevalence estimate should be interpreted cautiously. Second, only the English language was used to retrieve studies. Third, due to the paucity of study reports on HIV coinfection among VL patients outside of Northwest Ethiopia, our study was restricted to Northwest Ethiopia where it contributes over 60% cases of VL in Ethiopia. However, this study has several strengths that need to be mentioned: I) PRISMA guideline has been strictly followed, II) Large sample sizes of VL infected patients have been included to estimate pooled prevalence, III) Publication bias assessment and sensitivity analysis have also been conducted to ensure the robustness of study, IV) Evaluation of possible source of heterogeneity and trend analysis was also done.

## Conclusions

This study has some implications for practice and research. First, screening for HIV/AIDS should be performed among VL patients who live or travel to VL endemic regions. Second, visceral leishmaniasis and HIV/AIDS prevention and control strategies should be integrated and well-coordinated in areas where visceral leishmaniasis is endemic. Third, research should be carried out in VL endemic areas with no previous study report on the prevalence of HIV coinfection among VL infected individuals, particularly in Northeast and South-west Ethiopia.

## Data Availability

All data generated or analysed during this study are included in this published article (Table 1).

## Abbreviations

B: Bias coefficient
CI: Confidence interval
HIV: Human immunodeficiency virus
HIV-VL: Human immunodeficiency virus co-infection and visceral leishmaniasis
VL: Visceral leishmaniasis
